# Ectodermal dysplasias: New perspectives on the treatment of so far immedicable genetic disorders

**DOI:** 10.3389/fgene.2022.1000744

**Published:** 2022-09-06

**Authors:** Holm Schneider

**Affiliations:** Center for Ectodermal Dysplasias and Department of Pediatrics, University Hospital Erlangen, Friedrich-Alexander University Erlangen-Nürnberg, Erlangen, Germany

**Keywords:** ectodermal dysplasia, molecular therapy, ectodysplasin A, neonatal Fc receptor, stem cell, prosthodontic rehabilitation, tissue-engineering

## Abstract

The past decade has witnessed an expansion of molecular approaches facilitating the differential diagnosis of ectodermal dysplasias, a group of genetic diseases characterized by the lack or malformation of hair, teeth, nails, and certain eccrine glands. Moreover, advances in translational research have increased the therapeutic opportunities for such rare diseases, and new dental, surgical, and ophthalmic treatment options are likely to offer relief to many individuals affected by ectodermal dysplasias. In X-linked hypohidrotic ectodermal dysplasia (XLHED), the genetic deficiency of the signaling molecule ectodysplasin A1 (EDA1) may even be overcome before birth by administration of a recombinant replacement protein. This has been shown at least for the key problem of male subjects with XLHED, the nearly complete absence of sweat glands and perspiration which can lead to life-threatening hyperthermia. Prenatal treatment of six boys by injection of an EDA1 replacement protein into the amniotic fluid consistently induced the development of functional sweat glands. Normal ability to sweat has so far persisted for >5 years in the two oldest boys treated *in utero*. Thus, timely replacement of a missing protein appears to be a promising therapeutic strategy for the most frequent ectodermal dysplasia and possibly additional congenital disorders.

## Introduction

The genetic basis of the prenatal development of vertebrates has been studied extensively for almost five decades, leading to a more comprehensive understanding of the interplay between the many genes involved and the developmental windows in which their expression is required. In the field of medicine, this knowledge may have both diagnostic and therapeutic implications.

The ectoderm, one of three germ layers in the early embryo, gives rise to the central and peripheral nervous system, the neural crest cells, the tooth placodes, and the epidermis with its appendages. Impaired ectodermal development, thus, can result in a variety of congenital disorders, including 189 conditions initially classified as ectodermal dysplasias (Freire-Maia, 1971; Pagnan and Visinoni, 2014). Three molecular pathways, namely ectodysplasin A, Wnt/β-catenin and tumor protein 63 (p63) signaling, have been found to play major roles in the budding and morphogenesis of ectodermal derivatives (Mikkola and Thesleff, 2003; Mikkola, 2007; Harris et al., 2008; Cui et al., 2014). Almost all genes known to regulate the position, shape, or number of teeth or hairs have important functions in the mediation of cell communication, which is generally considered the most important mechanism driving embryonic development (Thesleff, 2003). Integration of genomics has been instrumental in explaining complex sequential interactions between epithelium and mesenchyme that direct ectodermal differentiation. This brought about a new classification system for ectodermal dysplasias (Wright et al., 2019; Peschel et al., submitted) based on the molecular pathways rather than just the anatomical structures affected. Grouping rare diseases according to shared characteristics of pathogenesis is expected to facilitate differential diagnosis as well as genetic counselling and becomes even more important with molecular therapies on the horizon.

Several research groups have used animal models to establish successful approaches to treating disorders of ectodermal development (e.g., Gaide and Schneider, 2003; Hermes et al., 2014; Watanabe et al., 2015; Jia et al., 2017a; Jia et al., 2017b). Although the pharmaceutical industry has remained reluctant to invest in such strategies, one of them, namely the prenatal replacement of missing EDA1 in children with the most prevalent ectodermal dysplasia, the X-linked hypohidrotic form (XLHED), is currently being evaluated in a worldwide clinical study (ClinicalTrials.gov identifier: NCT04980638). This trial constitutes the first attempt to investigate a pharmacotherapy of genetic disease in patients *in utero*. If successful, knowledge transfer to the treatment of other congenital disorders will be facilitated. For ectodermal dysplasias caused by variants of the gene *TP63* (encoding the transcription factor p63), the typical skin and corneal lesions may be amenable to cell therapy using genetically corrected autologous stem cells. Last but not least, small molecule drugs are being increasingly used to improve the outcome of prosthodontic treatment, the longevity of dental implants, results of the surgical repair of orofacial clefts, or ocular surface regeneration. Similar small molecule therapies might also be developed for the treatment of other manifestations of ectodermal dysplasias.

## Molecular therapy before birth

In hypohidrotic forms of ectodermal dysplasia, such as XLHED, the congenital absence of most if not all sweat glands (Schneider et al., 2011) causes the main clinical problem. Why is that? While exercising, for example, muscles produce heat. In response, heat sensors in the hypothalamus send a signal to the sweat glands that induces perspiration. The sweat subsequently evaporates from the skin, and this evaporation cools the body. Sweating is essential for our thermoregulation particularly during outdoor activities on sunny days, in cases of a febrile virus infections, or in hot environments. Young infants exposed to heat are at highest risk of developing a heat-related illness. If you place a newborn who cannot sweat in an incubator, his head will soon become red and warm to the touch, the respiratory rate will go up dramatically, but his body temperature will rise despite panting. Within a few hours a life-threatening situation may develop (Blüschke et al., 2010). An older child at risk of overheating will intuitively search for water, shade, or cold surfaces to cool himself down, but such opportunities may not be as readily available as needed. Being unable to sweat is life-threatening—from the hour of birth! Apart from hypo- or anhidrosis there are other cardinal symptoms of XLHED, such as missing or misshapen teeth which do not erupt on time. Oligodontia and heat intolerance are usually more severe in boys with XLHED than in affected girls because male patients have a single copy of the X chromosome that carries the disease-causing mutation, while in females the two X chromosomes most often differ in genetic content and the variant is usually expressed in only about half the cells. The absence of multiple teeth, however, allows non-invasive prenatal diagnosis as early as 5 months before birth by tooth bud counting during a routine fetal ultrasound scan (Wünsche et al., 2015; Hammersen et al., 2019).

### Clinical trials of postnatal protein replacement

Individuals with XLHED lack functional EDA1, a heterotrimeric transmembrane protein of the TNF family, the signaling part of which is released by the protease furin leading to its circulation with the blood stream. For more than 15 years an international research consortium has been investigating a recombinant replacement protein that resembles natural EDA1. In this molecule named Fc-EDA, the receptor binding domains are linked by Fc fragments of immunoglobulins. It binds to the natural EDA1 receptor (EDAR) and activates the signaling cascade inducing the formation of sweat glands, hair, and teeth. Fc-EDA has been evaluated in clinical trials: first in a Phase I study in adults with XLHED who received five doses of the drug intravenously (ClinicalTrials.gov identifier: NCT01564225). Two dose cohorts were investigated, in which no relevant side effects, except for the formation of anti-drug antibodies, were observed. Then we conducted a Phase 2 dose-escalation study in infants with XLHED who were treated within 28 days after birth (ClinicalTrials.gov identifier: NCT01775462), and we assessed safety, pharmacokinetics, and efficacy of Fc-EDA. Unfortunately, almost no sweat ducts could be detected by confocal laser-scanning microscopy after postnatal dosing and the subjects were unable to perspirate (Körber et al., 2020). Hence, the most important aim had not been achieved. We reasoned that, if there was any chance of success, we would need to administer the drug within the natural time window of sweat gland formation.

### Treatment with Fc-EDA *in utero*

**FIGURE 1:**
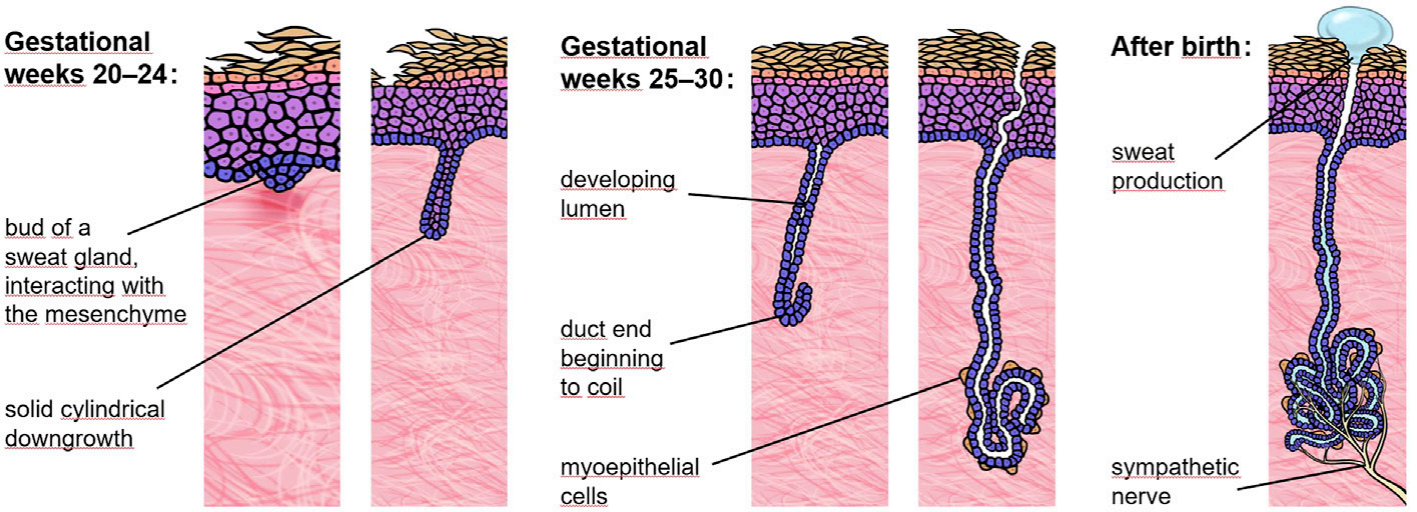
Eccrine sweat gland development. In the human fetus, eccrine sweat glands begin to form in palmoplantar skin and a bit later (around gestational week 20) across the rest of the body. At 30 weeks of gestation most sweat glands have already completed the critical portion of development but still lack sympathetic innervation.

In humans, eccrine sweat gland development starts around gestational week 20 with the formation of a placode in the epidermis. This placode soon undergoes solid cylindrical downgrowth into the mesenchyme (Figure 1). Once long enough, a lumen develops, the duct end begins to coil and becomes surrounded by myoepithelial cells. All these events take place in gestational weeks 20–30 (Ersch and Stallmach, 1999). Sympathetic innervation of the gland, however, is only completed postnatally. Thus, in order to rescue sweat gland formation, delivery of Fc-EDA at the end of the second trimester of pregnancy appeared to be the most rational approach. That brings us back to an essential component of our replacement protein, the Fc fragments, which allow a new drug-delivery method using the neonatal Fc receptor (FcRn). In mammals, including human babies, FcRn is situated in gut epithelia and mediates the uptake of maternal antibodies from breast milk. It binds the Fc portion of such antibodies and transports them across the intestinal epithelium into the baby’s blood (Martins et al., 2016). FcRn-mediated transcytosis is already operational in the fetus in the third trimester of pregnancy when a fetus regularly swallows amniotic fluid. In *Tabby* mice, a naturally occurring animal model of XLHED, prenatal administration of Fc-EDA into the amniotic cavity of affected embryos was shown to prevent the disease completely (Hermes et al., 2014). This treatment only corrected the development of hair, teeth, and sweat glands in the presence of intact FcRn, proving that the therapeutic route of the drug is systemic rather than through a direct effect on the skin and oral epithelia (Schneider et al., 2018). In dogs with XLHED, minimally invasive delivery of Fc-EDA into the amniotic fluid also rescued the formation of skin appendages and teeth (Margolis et al., 2019).

During this time we were approached by a pregnant woman with a family history of XLHED who was concerned that the twins she was carrying would be affected. Her older son had the disease and showed a complete absence of sweat glands and perspiration. Prenatal tooth germ counting led to the diagnosis of XLHED in both male twins. We investigated the functional impact of the *EDA* variant identified in the family, c.911A>G (p.Y304C), which was demonstrated to result in an insoluble EDA1 protein with complete loss of function and, thus, represents a null mutation. The parents requested compassionate use of Fc-EDA to treat the affected dichorionic twins *in utero*. This request was considered by our team and finally approved by the ethics committee of our university hospital. Using ultrasound guidance, we administered two doses of the drug into each amniotic cavity at gestational weeks 26 and 31. Postnatally, an intriguing finding was the rise in the number of tooth germs. After treatment *in utero* we detected 10 and 8 tooth buds, respectively, whereas prenatal sonography and MRI had revealed no more than two tooth germs in either twin (Schneider et al., 2018). The untreated older brother had three teeth and one additional tooth bud in total. Even more impressive was the normal sweat-duct density on the soles of the feet, while we did not observe any sweat ducts in the untreated older brother. The twins’ ability to perspire, quantified by measuring pilocarpine-induced sweat production, also proved to be normal. To date pilocarpine-induced sweating has remained at roughly the same level. No hyperthermic episodes or XLHED-related hospitalizations have occurred. And there are good reasons to assume that once sweat glands are normally formed they will work permanently.

Half a year later we treated another affected boy who received, however, only one dose of Fc-EDA *in utero*. This boy developed slightly fewer sweat glands and produced less sweat than the treated twins, suggesting an advantage of repeated dosing (Schneider et al., 2018). Although the replacement protein was detectable in cord blood samples, confirming an efficient uptake from amniotic fluid into the fetal blood, Fc-EDA serum concentrations in the pregnant women always remained below the detection limit, indicating minimal if any maternal drug exposure. Anti-drug antibodies that had been found after intravenous administration in adult males and non-pregnant females did not appear to be elicited in the mother when Fc-EDA was delivered intra-amniotically (Körber et al., 2020). Transillumination of the treated infants’ eyelids revealed a larger amount of Meibomian glands than usually present in subjects with XLHED (Schneider et al., 2018). In all three treated boys, we observed increased, basically normal saliva production. Thus, the prenatal therapy does not only rescue sweat gland development but also seems to have an impact on the function of other eccrine glands. Three more affected boys received Fc-EDA on a named-patient basis repeatedly between gestational weeks 25 and 31 and are now able to perspire as efficiently as the infants we had treated before.

The efficacy and safety of Fc-EDA (now also called ER004) as a prenatal pharmacotherapy for male subjects with XLHED is currently being investigated in a multicenter clinical trial, benefitting from the PRIME scheme of the European Medicines Agency and fast-track designation by the FDA. Up to 20 patients will be treated *in utero* with three injections of Fc-EDA, the first one at the beginning of gestational week 26. At the age of 6 months, the pilocarpine-induced sweat volume and other efficacy endpoints will be compared to data of untreated affected relatives.

## Generation of replacement tissues for therapies after birth

Ectodermal dysplasias resulting from a dysfunctional p63 protein, in particular ankyloblepharon-ectodermal dysplasia-cleft lip/palate (AEC) syndrome and ectrodactyly-ectodermal dysplasia-cleft lip/palate (EEC) syndrome, are characterized by typical tissue defects. Most of the children with AEC syndrome suffer from painful skin erosions that can lead to infection, scarring, and hair loss (Siegfried et al., 2005; Maillard et al., 2019). In patients with EEC syndrome, recurrent corneal lesions are frequently observed, culminating in loss of vision (Felipe et al., 2012). The primary cause of the slow corneal healing process appears to be a malfunction and later disappearance of limbal stem cells (Di Iorio et al., 2012). Orofacial clefts that are typical manifestations of both AEC and EEC syndrome require surgical treatment of soft tissue and bone defects. Patients with many types of ectodermal dysplasia, irrespective of the molecular pathway impaired, are afflicted with a lack of teeth. Preservation of oral function includes dental implant therapy, often associated with bone augmentation prior to implant placement.

### Regenerative therapies for skin and corneal lesions

In patients with AEC syndrome, treatment of chronic skin lesions based on the expansion and grafting of genetically corrected autologous keratinocytes might be feasible and has been discussed (Koch and Koster, 2021). This may involve the generation of patient-specific induced pluripotent stem cells, correction of the disease-causing *TP63* mutation with genome editing techniques, subsequent differentiation of corrected stem cells into keratinocytes, and production of keratinocyte sheets to be transplanted onto the chronic wound, a technically very challenging and time-consuming approach. Alternatively, such keratinocyte sheets could also be produced from patient-derived epidermal stem stells, as shown recently by the successful regeneration of almost an entire, fully functional epidermis on a child with severe junctional epidermolysis bullosa (Hirsch et al., 2017; Kueckelhaus et al., 2021). In this respect, the CRISPR/Cas genome editing system may hold a lot of promise. Novel engineered CRISPR-associated endonucleases allow for much higher specificity and fewer off-target effects than previous systems, which has improved gene editing for autosomal dominant diseases that cannot be tackled by alternative allele-specific gene therapy (Caruso et al., 2022).

Similar strategies might be used to treat non-healing corneal abrasions in patients with EEC or AEC syndrome. Human limbal stem cells, which are crucial for corneal epithelial regeneration and for the maintenance of a physical barrier between the clear, avascular cornea and the vascularized conjunctiva, can be cultured *in vitro* for transplantation (Rama et al., 2010). Autologous, *ex vivo* expanded epithelial cells containing limbal stem cells were approved some years ago as an advanced-therapy medicinal product to treat severe limbal stem cell deficiency (Pellegrini et al., 2018). Regeneration of the cornea with this method has led to the recovery of vision in numerous patients and is one of the first examples of a successful stem cell therapy (Pellegrini et al., 2018; De Luca et al., 2019). Considering the healthy marriage of gene and stem cell therapy in other fields (Nogrady, 2019), I see sense in merging the two also for the treatment of p63-associated syndromes.

In addition, the small molecule drug PRIMA-1^MET^, a p53 reactivator which has been tested in clinical trials for the treatment of malignant neoplastic disease, was recently shown to rescue epidermal differentiation and to improve wound healing in patients with AEC syndrome when applied topically to affected regions of the skin (Aberdam et al., 2020). Its mechanism of action, however, has remained unclear.

### New dental treatment options

Dental care of individuals with ectodermal dysplasias is challenging, but may profit tremendously from novel biomaterials and oral rehabilitation strategies. Treatment during childhood ranges from tooth shape corrections using composite build-ups to removable partial or complete dental prostheses and orthodontic appliances. Presurgical infant orthopedics and maxillofacial surgery are required for cleft palate repair (in case of p63-associated syndromes). If many secondary teeth are absent, orthodontic space closure as well as teeth distribution to improve prosthetic restoration will be helpful. This is often accompanied by maxillofacial surgery preparing the jawbone for dental implants. Alternatively, autogenous tooth transplantation may be considered.

Present-day prosthodontic rehabilitation concepts frequently include the replacement of at least some missing teeth by endosseous implants. As a result of hypo- or even anodontia, however, the bony ridge of the jaw that normally holds the teeth in place fails to develop properly. Therefore, bone grafting is a common prerequisite for implant surgery in patients with ectodermal dysplasias (Chrcanovic, 2018). Guided alveolar ridge augmentation involves the transplantation of bone from another site of the body, donor bone, or synthetic bone substitute that is covered with a semi-permeable membrane. Natural osteogenesis then leads to a new bony base for the implant (Chrcanovic, 2018). Stability and long-term survival of dental implants depend on their osseointegration. Various small molecules with osteoinductive activity have been investigated that induce for example bone morphogenetic protein-2 signaling or the Wnt pathway. Such small molecule drugs can be administered together with biomaterials and direct the differentiation of target cells, improve survival of the newly formed tissue in the body, or stimulate endogenous stem cells to enhance tissue repair (Lu and Atala, 2014).

**FIGURE 2:**
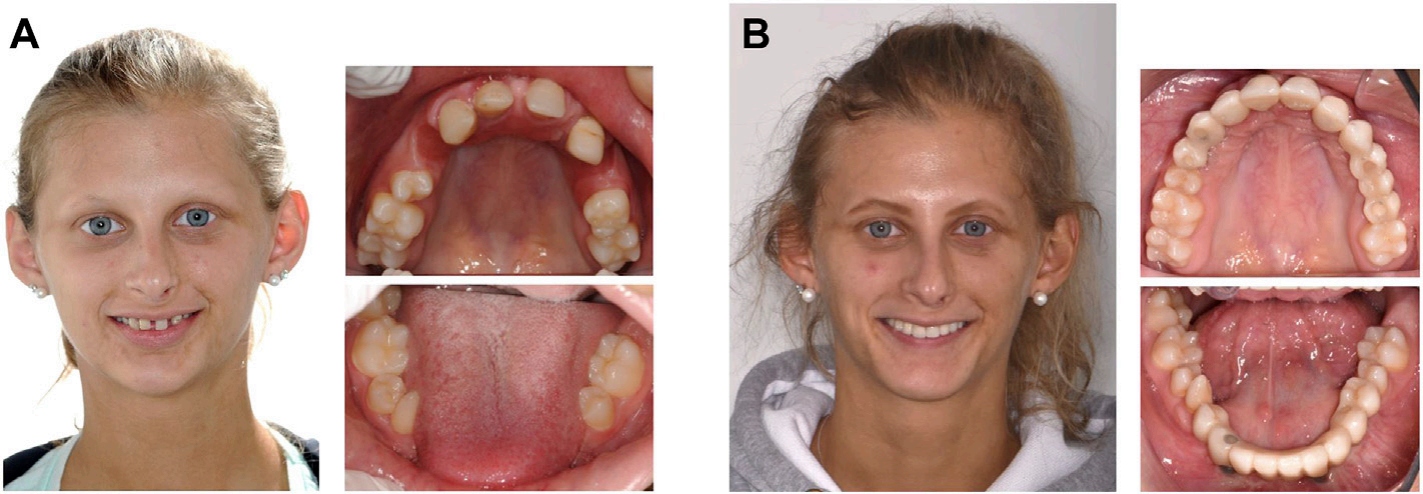
Oral rehabilitation in a woman with ectodermal dysplasia missing 14 permanent teeth. **(A)**, affected female adolescent before multidisciplinary dental treatment. **(B)**, results of prostodontic rehabilitation. (Photos: Prof. Dr. Stephan Eitner, Department of Prosthodontics, and Dr. Dr. Ines Willershausen, Department of Orthodontics and Orofacial Orthopedics, University Hospital Erlangen).

Today the outcome of multidisciplinary dental care is usually satisfactory and sometimes astonishing (Figure 2). This has contributed to increased self-esteem of children, adolescents, and adults with ectodermal dysplasias.

## Conclusion

Therapeutic perspectives for individuals affected by ectodermal dysplasias have become exciting. Drug targeting *via* the neonatal Fc receptor may cure a so far immedicable genetic deficiency: the life-threatening inability to sweat associated with XLHED. Such treatment is efficient *in utero*, but without significant therapeutic effects if performed after birth. A pivotal clinical trial of fetal therapy that could lead to an approval of the new drug is now recruiting patients. In this study, Fc-EDA will be administered to XLHED-affected male fetuses, trying to confirm the clinical improvements we have seen in the first six boys treated *in utero*. Last but not least, the same drug delivery route might be usable for the treatment of other disorders of early development. Translational research is also paving the way to more effective regenerative therapies for skin and corneal lesions using patient-derived epidermal or limbal stem stells. Furthermore, dental treatment options based on novel biomaterials, replacement of missing jawbone, and prosthodontic rehabilitation exist that will help improve oral function in almost all individuals with ectodermal dysplasias.

## Data Availability

Access to the original data of clinical trials is restricted to protect confidential or proprietary information. The data that support the findings reported here are available from the author upon reasonable request. Requests to access these datasets should be directed to Holm Schneider, holm.schneider@uk-erlangen.de.
